# Development and validity of a mentally-passive and mentally-active sedentary time questionnaire in nursing college students

**DOI:** 10.3389/fpubh.2023.1180853

**Published:** 2023-09-19

**Authors:** Meiling Qi, Yiming Gao, Xiangyu Zhao, Cindy Jones, Wendy Moyle, Shiyu Shen, Ping Li

**Affiliations:** ^1^School of Nursing and Rehabilitation, Cheeloo College of Medicine, Shandong University, Jinan, Shandong, China; ^2^College of Xinjiang Uyghur Medicine, Hetian, Xinjiang, China; ^3^Menzies Health Institute Queensland, Griffith University, Nathan Campus, Brisbane, QLD, Australia; ^4^Faculty of Health Sciences and Medicine, Bond University, Gold Coast, QLD, Australia; ^5^School of Nursing and Midwifery, Griffith University, Nathan Campus, Brisbane, QLD, Australia; ^6^Jinan Vocational College of Nursing, Jinan, Shandong, China

**Keywords:** sedentary behavior, nursing, mentally-passive, mentally-active, health promotion

## Abstract

**Objective:**

This study aimed to develop and validate a questionnaire to evaluate nursing college students’ mentally-passive and mentally-active sedentary time (M-PAST) in China.

**Methods:**

An initial M-PAST questionnaire with mentally-passive and mentally-active sedentary behaviors was developed with content validity undertaken through a consensus panel and pilot test where a convenience sample of six nursing students was recruited to assess the relevance, comprehensiveness, and comprehensibility of the refined questionnaire after expert panelists’ responses. A cross-sectional online survey using a self-reported questionnaire was distributed to nursing students by email and then conducted using exploratory factor analysis (EFA) and confirmatory factor analysis (CFA) to assess the construct validity of the M-PAST questionnaire and factor structures. Finally, the criterion validity was examined by exploring the associations between the M-PAST and the IPAQ sitting time, psychological distress, and insomnia.

**Results:**

Eight items regarding learning and leisure were included in the final version of the M-PAST questionnaire. A group of 650 nursing college students in China completed the study. Principal component analysis revealed two factors (i.e., mentally-passive and mentally-active sedentary behaviors), which explained 41.98% of the variance contributing to the questionnaire. The CFA reached the adaptive standard. Cronbach’s *α* ranged from 0.730 to 0.742. The correlations between M-PAST and IPAQ total sitting time were significant (*p* < 0.01, *r* = 0.125–0.396). Mentally-passive sedentary time was associated with psychological distress and insomnia (*p* < 0.01, *r* = 0.078–0.163), while no significant associations were found in mentally-active sedentary behaviors.

**Conclusion and implications for practice:**

The M-PAST questionnaire appears to be a reliable and valid tool that reported both mentally-passive and mentally-active sedentary behaviors in nursing college students in China. However, future studies may need to further examine its validity among international nursing college students. This study further confirmed that mentally-passive sedentary behavior was positively associated with psychological distress and insomnia. Effective strategies are needed to reduce nursing college students’ mentally-passive sedentary time to improve their health and wellbeing in China.

## 1. Introduction

Nursing college students were reported to experience prolonged sitting time during the COVID-19 pandemic by more than 50% compared to pre-COVID-19 (1). Uninterrupted classroom sitting was associated with increased discomfort and sleepiness in nursing college students (2). Prolonged sitting is also strongly associated with low levels of physical activity, psychological distress, and insomnia, resulting in academic, interpersonal, and functional challenges (3–5). Many studies reported that nursing students experienced a high prevalence of unhealthy lifestyles, including sleep deprivation and psychological disorder, in Brazil (6), India (7), and China (8), where sedentary behavior/physical inactivity are considered to be potential factors that lead to an unhealthy lifestyle. Belingheri et al. (9) also found that nursing students reported a higher prevalence of sleep disorders partially because of physical inactivity/sedentary behavior than students in other subjects. Given that nursing college students spend most of their weekdays in classroom environments that leads to prolonged sitting, reducing sedentary time in nursing college students could be a health promotion strategy to reduce their symptoms of psychological distress and insomnia. Although prolonged sedentary time was reported to be a potential risk factor for multiple adverse health outcomes (e.g., psychological distress and insomnia), diverse sedentary behaviors may have inequivalent detrimental effects on these health outcomes (10). This study develops and validates a questionnaire to evaluate nursing college students’ mentally-passive and mentally-active sedentary time in China, and explores their associations with psychological distress and insomnia.

Sedentary contexts are often divided into three broad categories: occupation/learning, leisure, and transport referring to where the sedentary behaviors usually occur (11, 12). Furthermore, Werneck et al. (13) described diverse sedentary types, including mentally-passive and mentally-active sedentary behaviors according to the nature of the activity itself. For example, TV viewing is a type of mentally-passive sedentary behavior as it requires low cognitive demand and is usually undertaken in leisure. In contrast, work-related tasks and reading books involves concentration and cognitive effort. Thus, occupational/learning sedentary behaviors would logically be considered mentally-active (11). As for the effects of mentally-passive and active sedentary behaviors, studies found that mentally-passive sedentary behaviors appear to increase the risk of depression and cognitive impairment in older adults and adolescents, whereas mentally-active sedentary behaviors may protect against these outcomes (11, 13, 14). However, limited studies have examined the effects of different types of sedentary behaviors on the health status of nursing college students due to the lack of a valid questionnaire to examine mentally-passive and active sedentary sitting time in this population.

The preliminary framework to guide the development of sedentary behavior questionnaires also suggested assessing sedentary behaviors across three contexts (i.e., occupation/learning, leisure, and transport) and two types (i.e., mentally-passive and mentally-activity) (11). While existing studies commonly used the international physical activity questionnaire (IPAQ), this is limited to only one item that assesses the total sitting time of the past 7 days (15, 16). Mentally-passive and mentally-active sedentary questionnaires found that most studies used one or two questions to assess the nature of the sedentary behaviors (12, 13). They reported no established validity and reliability, especially for use by college students. Moreover, Other existing questionnaires, for example, the sedentary behavior questionnaire (SBQ) (17) and the adolescent sedentary activity questionnaire (ASAQ) (18), assessed the time spent in sedentary activities in more than five domains (e.g., playing computer games, reading, relaxing with friends, and going to church). In addition, the sedentary sitting time recorded devices (e.g., activPAL3 activity monitor) have been used to objectively record individuals’ sitting time in a college context (19). However, these questionnaires and objective sedentary behavior measures do not differentiate sedentary behaviors into specific contexts and categories of either mentally-passive or mentally-active. In addition, not all the included items were appropriate for nursing college students and no specific college student questionnaire has been developed.

There has been a suggestion that besides the assessment of total sedentary time, a different approach to the assessment of sedentary sitting is needed where both the contexts and types of sedentary behaviors should be considered (11). Therefore, developing an appropriate and valid assessment tool, which delineates between mentally-passive and mentally-active sedentary behaviors with three contexts and the time spent on these behaviors, is necessary to understand how different types of sedentary behaviors associate with or explain the health status (i.e., psychological distress and insomnia) of nursing college students. In addition, it is of great importance to design such a questionnaire for recording both mentally-active and mentally-passive sitting time separately which cannot be achieved by objective sedentary behavior measures. Thus, the current study aimed to explore nursing college students in China to (a) develop a self-reported mentally-passive and mentally-active sedentary time (M-PAST) questionnaire, (b) test the content and construct validity of the M-PAST questionnaire as well as its criterion validity using the associations between the M-PAST and the IPAQ sitting time, psychological distress, and insomnia, and (c) test the reliability of the M-PAST questionnaire.

## 2. Methods

### 2.1. Phase 1: development of the M-PAST questionnaire

The development and content validity of the M-PAST questionnaire was undertaken using a consensus panel and pilot test. Expert panelists’ feedback was summarized to inform changes until group consensus among experts was achieved. A pilot test was then conducted among a convenience sample of six nursing students to assess the relevance, comprehensiveness, and comprehensibility of the refined M-PAST questionnaire (i.e., 8 items) based on experts’ responses. Ethics approval was received from the Research Ethics Committee of the affiliated institute (2021-R-164).

#### 2.1.1. Preliminary item selection

An initial self-reported M-PAST questionnaire comprising 10 items in three contexts (i.e., learning, leisure, and transport) was developed for nursing college students in China, including (1) learning, (2) reading books/newspapers, (3) socializing, (4) playing electronic games, (5) watching TV/films/videos, (6) browsing websites/moments/online shop, (7) having meals, (8) napping, (9) sitting as a passenger in a car, and (10) driving a car. Sedentary behaviors were defined by the energy expenditure of <1.5 metabolic equivalents (METs) (e.g., equivalent to sitting or lying down) (20). In addition, based on differential concentration and cognitive effort/demand of different sedentary behaviors, the items (1), (2), (3), (4), and (10) are characterized by cognitive effort. These are referred to as mentally-active sedentary behaviors, with the remaining five items being mentally-passive sedentary behaviors as they involve passive mental activity.

Participants were asked, “how often do you perform the above certain activity while sitting or lying down on weekdays and weekends separately during the past week? For each activity, please respond with the approximate duration per day you perform the activity on weekdays and weekends separately.” The total duration of mentally-passive and mentally-active sedentary sitting time on weekdays and weekends in the past week was calculated.

#### 2.1.2. Refinements based on responses from experts

Content validity of the M-PAST content was undertaken using a consensus panel between 15 May and 30 July 2021. This panel comprised six nursing, public health, and physical health professors recruited from the university by word of mouth and email. Expert panelists were health professors experienced with exercise or nursing researchers interested in health promotion. After recruitment, potential expert panelists were provided a study information sheet by email that explained the purpose of the study, study risks and benefits, the refinement process, and contact information. Consenting expert panelists were asked to sign and email their written informed consent forms back to the researchers. Expert panelists then received the M-PAST questionnaire via email and adjusted whether the item was relevant on two occasions. For each item, a score of 1 to 5 points was assigned, 5 very relevant, and 1 irrelevant. Items were discarded if an average total of <2 points was obtained (21).

The original two questionnaire items (i.e., items 9 and 10) about transport context (with an average of 1.33 points) were found to be not suitable for Chinese college students due to on-campus residency in China (22). Finally, eight items were included within two contexts (i.e., learning and leisure) with the items (1), (2), (3), and (4) referring to mentally-active sedentary behaviors and the remaining four items (i.e., 5, 6, 7, 8) being mentally-passive sedentary behaviors.

#### 2.1.3. Pilot test

Following refinements of the M-PAST questionnaire (i.e., 8 items) based on experts’ responses, a pilot test was conducted between 15 August and 30 August 2021 by asking a convenience sample of six nursing students (i.e., ages ranging from 20 to 22 years) using an online survey administered via a free online Chinese survey platform^1^ where two of them were males and four were females. The ten criteria for good content validity of a patient-reported outcome measure (PROM) were used to assess the relevance (five criteria), comprehensiveness (one criterion), and comprehensibility (four criteria) of the refined M-PAST questionnaire (23). No items were excluded from the final version of the revised M-PAST questionnaire after the pilot test (see Table 1).

**Table 1:** Items in the developed M-PAST questionnaire.

Sedentary contexts	Questionnaire items	Sedentary types	Questions for each item
Learning	1. Learning	Mentally-active	How often do you perform the activity while sitting or lying down on weekdays and weekends separately during the past week? Please record the approximate duration per day you perform the activity on weekdays and weekends separately
Leisure	2. Reading books/newspapers		
	3. Socializing		
	4. Playing electronic games		
	5. Watching TV/films/videos	Mentally-passive	
	6. Browsing websites/moments/online shop		
	7. Having meals		
	8. Napping		

### 2.2. Phase 2: validity of the M-PAST questionnaire

#### 2.2.1. Design

A cross-sectional study using an online survey design was performed to assess the construct and criterion validity of the developed M-PAST questionnaire. Construct validity indicates the degree to which the instrument is consistent with hypotheses (i.e., mentally-active and mentally-passive sitting time) (24). Criterion validity is defined as the degree to which the scores of an instrument are an adequate reflection of the “gold standard” and are positively or negatively associated with the scores of other instruments (25, 26). The current study examined the agreement between the sitting time of the IPAQ and the developed M-PAST. Although the IPAQ is not a “golden standard,” the IPAQ has already been validated in Chinese language and college students (27) and so the sitting time of IPAQ was used to support the criterion validity of the M-PAST questionnaire in the current study. In addition, the M-PAST sitting time should correlate with psychological distress and insomnia. Their associations were also used to assess the criterion validity of the M-PAST questionnaire.

This study was conducted following the Declaration of Helsinki established by the World Medical Association and is reported according to the STROBE reporting guidelines for cross-sectional studies. Ethics approval for this validation study was received from the Research Ethics Committee of the affiliated institute (2021-R-165). Completion and submission of the online survey implied consent to participate. This was declared to respondents at the commencement of the survey.

#### 2.2.2. Participants and recruitment

Using convenience sampling, nursing students from a large medical college in China were invited to participate. An email invitation with the help of the College Deputy Vice Administration Office was sent to all nursing students. The email invitation included the purpose of the study, inclusion and exclusion criteria, and the online survey link (see footnote 1) to the M-PAST questionnaire.

#### 2.2.3. Data collection

Data collection took place between 10 October and 30 December 2021. The study survey included participants’ demographic details (i.e., age, gender, and year of study), the M-PAST questionnaire, IPAQ-short form (IPAQ-SF), and an assessment of psychological distress and insomnia.

The IPAQ-SF Chinese version was used to assess participants’ physical activity participation and average sitting time on weekdays and weekends during the past 7 days. There are two types of IPAQ scores for data processing and analysis: a categorical and a continuous score. The categorical score classified participants into three physical activity intensity levels (i.e., low, moderate, and high). The continuous score is expressed as the metabolic equivalent task (MET-minutes per week) of energy expenditure. In addition, participants sitting time (i.e., hours per day) was also recorded on the IPAQ-SF. High validity and reliability for the IPAQ-SF have been established among Chinese adults with intraclass correlation coefficients above 0.84 (28).

The 10-item Kessler psychological distress scale (K10) Chinese version was used to assess psychological distress. The K10 is a self-reported questionnaire containing 10 questions with a score ranging from 1 to 5 to assess participants’ frequency of nonspecific psychological distress across the past month based on questions related to symptoms of anxiety and depression. Participants choose how often they felt or thought in a certain way: 1 = almost never, 2 = sometimes, 3 = fairly often, 4 = very often, 5 = all the time. The total score was obtained by summing all 10 items, with a total score of 10–50. A score of 22 or greater indicates a high level of psychological distress. Higher scores indicate higher levels of psychological distress. The K10 scale is a valid instrument with acceptable internal consistency, with Cronbach’s *α* over 0.84 in adults over 18 years old (29).

The insomnia severity index (ISI) was used to assess participants’ insomnia symptoms. The ISI is a self-reported questionnaire containing seven questions with a score from 0 to 4 to assess participants’ degree of insomnia during the past week. The total score was obtained by summing all seven items, with a total score of 0–28. High scores indicate a higher degree of insomnia. A score of seven or less reflects no insomnia, with mild insomnia scores ranging from 8 to 14, moderate insomnia scores ranging from 15 to 21, and high insomnia scores ranging from 22 to 28. High validity and reliability for ISI have been established (Cronbach’s *α* = 0.91) for people over 18 years old (30).

#### 2.2.4. Statistical analysis

Data analysis was conducted using the Statistical Package for the Social Sciences (SPSS) version 25.0 and the AMOS 24.0 (IBM Corporation). Based on the data cleaning rules for the IPAQ-SF, respondents who reported over 960 min of total sedentary time per day were identified as over-reporting. The assumption is that individuals spend an average of 8 h of sleep per day (31). Descriptive statistics were calculated using frequencies (i.e., percentages) for categorical variables and mean and standard deviations for continuous variables.

The sample of 650 participants was randomly divided into two groups to investigate construct validity using exploratory factor analysis (EFA) (*n* = 325) and confirmatory factor analysis (CFA) (*n* = 325). The EFA was conducted using the principal component analysis (PCA) and varimax rotation to provide evidence of a stable factor structure. The PCA was conducted when the KMO was over 0.06, and the Bartlett test of sphericity was significant (*p* < 0.05). Items with factor loading >0.4 significantly contributed to a factor (21). Analysis of eigenvalues in the screen plot and the commonly applied eigenvalue criterion (>1.0) were used to determine the number of factors remaining for the final questionnaire. AMOS (IBM Corporation) was used to perform the CFAs of the M-PAST questionnaire, analyzing the fit of models of its respective parameter estimates. Additionally, the criterion validity of the M-PAST questionnaire was investigated using the Spearman correlation coefficients (*r*) to determine the correlation between the M-PAST and the IPAQ sitting time, psychological distress, and insomnia.

Cronbach’s *α* was used to evaluate the internal consistency, with Cronbach’s α being acceptable for values >0.7 (21). An independent *t*-test and Spearman correlation coefficients (*r*) were used to separately evaluate differences in the mentally-passive and mentally-active sedentary factors among different sexes and ages. The significant level was set at 0.05.

## 3. Results

### 3.1. Participants’ characteristics

Six hundred fifty nursing college students were included for analysis in this study after removing those who reported more than 960 min of total sedentary time per day (*n* = 85; 11.6%). The majority of participants were female (*n* = 506; 77.8%) (see Table 2), 27.7% were first-year college students, with 36.9% and 35.4% participants being second-year and third-year participants, respectively. Almost half of the participants engaged in moderate physical activity levels, and only 21.5% reported high-intensity activity.

**Table 2:** Demographic characteristics of the participants (*n* = 650).

Variables	$\bar{x}$± *s*/*n*	Range/%
Age	19.52 ± 1.08	17~23
*Gender*		
Male	144	22.2%
Female	506	77.8%
*Year of study*		
First-year	180	27.7%
Second-year	240	36.9%
Third-year	230	35.4%
*IPAQ levels*		
Low intensity	191	29.4%
Moderate intensity	319	49.1%
High intensity	140	21.5%

IPAQ, international physical activity questionnaire.

### 3.2. Construct validity analysis

The KMO criterion was acceptable at 0.665, and the Bartlett test of sphericity was significant (χ^2^_190_ = 254.656; *p* < 0.001). Using PCA and varimax-rotation as the extraction methods, the scree-plots revealed two factors that accounted for 41.98% of the variance. Table 3 presents the factor-loading matrix of the eight items, consistent with the original two-factor assumption. The first factor of the two-factor-solution consisted of four items (i.e., 1, 2, 3, and 4) with high loadings and accounted for 21.68% of the variance in the explained model (eigenvalue = 2.18). These items refer to mentally-active behaviors with cognitive effort (e.g., learning). Thus, this factor was named “mentally-active sedentary behaviors.” Additionally, the second factor consisted of the four remaining items (i.e., 5, 6, 7, and 8) and accounted for 20.30% of the variance in the explained model (eigenvalue = 1.18). This factor reflects passive mental activity (i.e., watching TV) and is named “mentally-passive sedentary behaviors.”

**Table 3:** Factor-loading matrix and Cronbach’s *α*.

Factor and question number	Factor loadings
*FACTOR 1* ^a^	
1. Learning	0.816
2. Reading books/newspapers	0.618
3. Socializing	0.497
4. Playing electronic games	0.477
*FACTOR 2* ^b^	
5. Watching TV/films/videos	0.586
6. Browsing websites/moments/online shop	0.732
7. Having meals	0.633
8. Napping	0.528

a Cronbach’s *α* = 0.742.

b Cronbach’s *α* = 0.730.

The CFA indicated a good fit for a two-factor model. In the model fitness index, the chi-square degree of freedom was 1.199, the goodness-of-fit index was 0.984, the adjusted goodness-of-fit index was 0.968, the incremental fit index was 0.981, Tucker Lewis index was 0.969, the comparative fit index was 0.980, and the root mean square error of approximation was 0.025.

### 3.3. Criterion validity analysis

The correlations between the M-PAST and IPAQ total sitting time were significant on weekdays and weekends (*p* < 0.01, *r* = 0.125–0.396). The K10 scores were significantly related to mentally-passive sedentary time on weekdays (*p* < 0.01, *r* = 0.125) and weekends (*p* < 0.01, *r* = 0.163), indicating that longer mentally-passive sedentary time was correlated with negative mental health. Similarly, longer mentally-passive sedentary was also correlated with insomnia on weekdays (*p* < 0.05, *r* = 0.078) and weekends (*p* < 0.05, *r* = 0.097). No statistically significant associations were found between mentally-active sedentary behaviors and psychological distress or insomnia (Table 4).

**Table 4:** Criteria validity of M-PAST questionnaire (*n* = 650).

Variables	TST (weekdays)	TST (weekends)	M-AST (weekdays)	M-AST (weekends)	M-PST (weekdays)	M-PST (weekends)
IPAQ-ST (weekends)	0.084^*^	0.125^**^	0.078^*^	0.169^**^	0.072	0.033
IPAQ-ST (weekdays)	0.396^**^	0.121^**^	0.394^**^	0.154^**^	0.192^**^	0.055
K10	0.015	0.129^**^	−0.049	0.017	0.125^**^	0.163^**^
ISI	−0.005	0.027	−0.055	−0.049	0.078^*^	0.097^*^

^*^p < 0.05, ^**^p < 0.01, M-AST, mentally-active sedentary time; M-PST, mentally-passive sedentary time; TST, total sedentary time calculated by summing the M-AST and M-PST; IPAQ-ST, international physical activity questionnaire sitting time.

### 3.4. Reliability analysis

Internal consistency for the questionnaire was assessed by Cronbach’s *α* (Table 2). The total M-PAST questionnaire’s Cronbach’s *α* was 0.808, with 0.742 and 0.730 for mentally-active and passive sedentary behaviors, respectively.

Data on different ages and genders were pooled to confirm the two patterns of sedentary behaviors further. There are significant differences on the mentally-active sedentary time and mentally-passive sedentary time on weekdays (*p* < 0.05, *r* = − 0.12 to −0.27) among various ages. No significant differences were found in the weekdays’ mentally-passive and mentally-active, sedentary time among females and males. However, the mentally-active sedentary time on weekends was significantly shorter in females than in males (*p* < 0.001), whereas the mentally-passive sedentary time of females was significantly longer (*p* = 0.004) (Table 5).

**Table 5:** Mean scores in males and females (*n* = 650).

	Females (*n* = 506)		Males (*n* = 144)		*p*
	Mean	SD	Mean	SD	
M-AST (weekdays)	24.97	16.52	25.81	16.59	0.593
M-AST (weekends)	9.02	4.79	10.87	5.11	<0.001
M-PST (weekdays)	14.87	7.86	13.47	7.26	0.055
M-PST (weekends)	8.88	4.63	7.85	3.48	0.004

M-AST, mentally-active sedentary time; M-PST, mentally-passive sedentary time.

## 4. Discussion

To our knowledge, this is the first study that has developed a questionnaire for assessing various sedentary times within different contexts and examined its validity among nursing college students in China. This study presents an M-PAST questionnaire (i.e., 8 items) that assesses two types of sedentary behaviors: mentally-passive and mentally-active, sedentary behavior within learning and leisure contexts. However, two items of the transport context were excluded from the preliminary M-PAST questionnaire (i.e., 10 items) following experts’ review, as nursing college students are not used to driving a motor vehicle or sitting as a passenger in China. One reason was that nursing college students aged from 18 to 22 years old are usually characterized as holding limited disposable income and unlikely to have car ownership (32). In addition, unlike college students in other countries, who may be residing off-campus and travelling to university, college students in China are more likely to live in an on-site dormitory (22). Hence, they have fewer opportunities to drive or take a car. However, the revised M-PAST questionnaire may limit its use internationally, which suggests that future studies may need to add the two transport items and further examine its validity among international nursing college students.

This study outcome is comparable to other construct validation studies (21, 33). Factor analysis confirmed the two-component factor structures. Four questions (e.g.,1–4) identified mentally-active sedentary behaviors, including questions about sitting/lying down and “learning” or “playing electronic games” or “reading books/newspapers” or “socializing,” which need cognitive activities. No significant associations existed between mentally-active sedentary behavior, psychological distress, and insomnia. This result was inconsistent with previous studies where mentally-active sedentary behavior is suggested to have the potential of protection against mental disorders, as mentally-active sedentary time may be linked to nursing college students’ mental stimulation that improves mental health (12, 13).

Additionally, the positive effects of mentally-active sedentary behavior may be attributed to the increased cognitive demand and brain connectivity during mentally-active sedentary activities, which are also associated with mental health (13). Specific reasons for these differences may be attributed to methodological discrepancies between studies. For example, the different ages of participants may contribute to the different results, with most previous studies including adolescents from 11 to 16 years (13). There are also differences in sample sizes where there were more than 7,124 participants in previous studies (12, 13) compared to our study of only 650 participants. These findings suggest that a large sample size should be considered in future studies to find the significant effects of mentally-active sedentary behavior on the mental health of nursing college students.

The remaining four questions inquiring about mentally-passive sedentary behaviors included items about sitting/lying down and “watching TV/films/videos,” “browsing websites/moments/online shop,” “having meals,” or “napping.” This study observed statistically significant associations between mentally-passive sedentary time and psychological distress as well as insomnia, where longer mentally-passive sedentary time may be a risk factor for psychological distress and insomnia, which are in line with previous studies (11, 34–36). One apparent reason to explain this result may be the reduction of time engaged in physical activity, well-established effective prevention and treatment for mental health (37). A potential mechanism suggested that mentally-passive sedentary behavior like watching TV can hinder direct communications between individuals, reducing social interaction and increasing the potential for psychological distress (35). In addition, mentally-passive sedentary behavior like using a computer increased screen exposure which was linked to delayed bedtime, reduced sleep duration, and poor sleep quality (38). The mentally-passive sedentary behavior and its consequence on sleep quality may become prevalent in mental disorders, increasing anxiety, depression, and stress symptoms. Therefore, practical interventions aimed at raising awareness about negative health implications and training in behavioral self-regulation may be needed to reduce nursing college students’ mentally-passive sedentary time and improve health wellbeing (39, 40).

The Cronbach’s *α* and the two-factor internal-reliability had an acceptable value compared with similar studies (21, 41). This study showed that males tend to have longer mentally-active sedentary time than females on weekends. In comparison, females showed longer mentally-passive sedentary time than males on weekends. Deep-rooted gender differences may contribute to significant differences in sedentary time of females and males on weekends, including different psychosocial factors (e.g., self-perception of health, satisfaction with body image) (42) and motiving factors to engage in physical activity (42). Moreover, various purposes of the internet/devices may contribute to longer mentally-active and passive sedentary among males and females, as males are more likely to play online games (i.e., mentally-active sedentary activity) (43). In contrast, females use the internet/device for website browsing (i.e., mentally-passive sedentary activity) (44). In addition, there are significant differences in the level of physical activity between males and females (45), indicating that male students reported better physical activity self-efficacy than females, which contributes to high levels of physical activity engagement in male students. The differences in physical activity participation may also be one potential reason for the detected differences in the mentally-passive and mentally-active sitting time of different genders on weekends. Hence, it is important for future studies to look at interventions targeting reductions in nursing college students’ mentally-passive sedentary sitting times, for example, changing behaviors of physical activity participation and internet usage, particularly in female students.

### 4.1. Study limitations

Some limitations of this study should be considered. First, the lack of data collection at two-time points does not allow interpretation of test-retest reliability analysis. Assessing test-retest reliability is an area for improvement in future studies. Second, the criterion validity assessment was not measured by comparing it with a “gold standard” (e.g., activPAL3 activity monitor) which could provide perfectly true sedentary time, but using the agreement with the sitting time of the IPAQ and the associations with psychological distress and insomnia. The missing collection of sitting time using a “gold standard” is another limitation of this study. Third, the reported estimate of sedentary behavior and time may not accurately reflect the time spent on the various sedentary behaviors because of possibly multi-tasking and overlapping time. For example, nursing college students may sit in front of a computer for learning and leisure, so the time reported for “learning,” “reading,” and “watching TV” may overlap. Forth, using self-reported measures of the M-PAST may also be a study limitation, as self-reported outcomes can lower the accuracy of the data and further reduce the internal validity of the M-PAST questionnaire. Future studies with nursing college students could combine alternatively objective outcome measures (e.g., activPAL3 activity monitor) when using the M-PAST questionnaire to eliminate possible self-reported bias. Fifth, the current study is a convenience sample of nursing college students, and is not representative of college students. Finally, the gender distribution, with most respondents being females, also limits the generalizability of the study results.

## 5. Conclusion and relevance for clinical practice

Preliminary evidence demonstrates that the M-PAST questionnaire is a reliable and valid self-reported questionnaire to identify mentally-passive and mentally-active sedentary behaviors among nursing college students in China. Not only have content, construct, and criterion validities been established, but an acceptable internal consistency of the two factors structure in the M-PAST questionnaire has also been found. Mentally-passive sedentary behaviors are significantly and positively associated with both psychological distress and insomnia among nursing college students but not mentally-active sedentary behavior. Future studies should focus on the test-retest reliability assessment in a larger sample size. These findings suggest that practical strategies to reduce nursing college students’ mentally-passive sedentary sitting time are needed to promote their health and well-being.

The M-PAST questionnaire is a valid and reliable tool to assess nursing college students’ mentally-passive and mentally-active sedentary time in China. Importantly, the current study further confirmed that mentally-passive sedentary behavior was positively associated with psychological distress and insomnia. These findings can be useful for assessing the mentally-active and mentally-passive sedentary time of nursing college students, which can be used for the implementation of practical strategies for future nurses to reduce their mentally-passive sedentary sitting time and further promote their health and well-being.

## Data availability statement

The raw data supporting the conclusions of this article will be made available by the authors, without undue reservation.

## Ethics statement

The studies involving humans were approved by the Research Ethics Committee of the author’s institution. The studies were conducted in accordance with the local legislation and institutional requirements. The participants provided their written informed consent to participate in phase 1 of this study. Written informed consent was not obtained from the individuals in phase 2 of this study as completion and submission of the online survey implied consent to participate. This was declared to respondents at the commencement of the survey.

## Author contributions

MQ: conceptulization, data curation, formal analysis, writing—original draft, and writing—review and editing. YG: conceptulization, data curation, formal analysis, and writing—original draft. XZ: conceptulization, data curation, formal analysis, and writing—review and editing. CJ: data curation, data interpretation, and writing—review and editing. WM: data interpretation and writing—review and editing. SS: conceptulization, writing—review and editing, visualization, supervision, and project administration. PL: writing—review and editing, visualization, supervision, and project administration. All authors contributed to the article and approved the submitted version.

## Funding

The study was supported by the National Nature Science Foundation of China (grant numbers: 82272923) and the Shandong Province Social Science Planning Foundation (grant number: 21CSZJ30).

## Conflict of interest

The authors declare that the research was conducted in the absence of any commercial or financial relationships that could be construed as a potential conflict of interest.

## Publisher’s note

All claims expressed in this article are solely those of the authors and do not necessarily represent those of their affiliated organizations, or those of the publisher, the editors and the reviewers. Any product that may be evaluated in this article, or claim that may be made by its manufacturer, is not guaranteed or endorsed by the publisher.

## Footnotes

^1^ https://www.wjx.cn
